# Puffing of Doctors by the Newspapers

**Published:** 1868

**Authors:** 


### Puffing of Doctors by the Newspapers.

By reference to the report of the proceedings of the Atchison County Medical Society, it will be seen that body has passed a rssolution denying the right of the editors of newspapers to use the names of physicians, in their reports of accidents and cases in general, without the consent of the physician previously obtained. We heartily indorse this action, though we have no doubt, when the reportorial fraternity come to hear of it, they will find in it new evidence of the proscriptive and illiberal spirit of the medical profession. The barbarians of the outside world are utterly oblivious of that fine feeling of ethical justice which is possessed by every true gentleman of our profession. Hence they are not slow to censure a physician who politely declines to become a party to the bad treatment of another of his cloth. They cannot understand why they are not at liberty to bring in a new attendant in a case of sickness, without notification of the desire to change, to the old one—although the latter may have far more ability to conduct the case than the former, and be doing all that human knowledge could suggest, at the very time.

So when a man gets knocked down in the street, and Dr. —— is called to render his assistance, the reporters think it strange they should not be indulged in the privilege of a sensational article, in which the name of Dr. —— shall figure prominently, as being the means, not through Providence, but his own extraordinary abilities, of having rescued his fellow-citizen from an untimely death. They do not consider, for a moment, that they know no more of the merits of the case than they do of the nosology of diseases in general; that even if Dr. —— has pursued the intelligent course which a thorough knowledge of his profession points out, he has done no more, perhaps, than dozens of others *within the same community* could do; and that by thus singling him out as an object of eulogium, a downright injustice is done to his peers, his betters, and even to the people themselves, in thus investing him with an exclusive skill which he does not, in ninety-nine cases out of one hundred, possess. No true physician desires this kind of notoriety. If he is really learned in his profession, and is possessed of more than ordinary ability, he knows where to display it, that he may reap a fame above the suspicion of having purchased it—that is, through the organs of his profession.

But to do the reporters justice in these particular cases, we must admit that we have seldom seen these unfair puffs concerning physicians, except the doctor has himself had a hand in it. It is true that in recording a casualty in the daily papers, it is generally stated as a fact pertinent to its relation, that Dr. —— dressed the man's wound, or extracted the ball; but so long as the mere fact is stated as an item of news, and no effort is made to pay a glowing tribute to the doctor's skill, we do not know that we could find much fault with it, or stop the practice if we did. It might result as did the case of an over-sensitive friend of ours, who, observing his name mentioned in a paper, as being in attendance upon a man who had recently been hurt, excitedly called upon the editor to forbid his using his name publicly again. The editor, mistaking the nature of his offending, and anxious to rectify his error, stated the next morning, that our friend Dr. —— was not attending the case at all. This brought the doctor once more to the editor's sanctum, and he was informed that the apology was worse than the offense, inasmuch as it contained a falsehood. The obliging editor said in his next paper, that he was mistaken in stating in his previous issue that Dr. —— was not in attendance upon the man recently hurt; that the doctor, after first denying it, had now acknowledged that he was in attendance; and from the fact that the man's death had just been announced, he, the editor, was inclined to believe the doctor's last assertion to be the true one.

From our own observation, the irrelevant portion of newspaper articles referring to physicians, as we have before said, is most usually—not always, of course—prompted by the doctor or his agents. That this is true, will appear from the character of the article itself. This, apart from the mention of a physician in attendance upon an emergency, generally consists of a notice of a particular operation performed by the talented Dr. Marvelous; and it is really amusing to observe how small an achievement is sometimes made the basis for a huge display of fanfaronade. We remember well a local notice which appeared, a few years since, in one of our city papers, stating that Dr. —— had, upon the day before, performed a most difficult surgical operation—that of *extracting a needle* from beneath the skin of a woman's chest; and adding that the result was no less gratifying to the numerous friends of the doctor than it was to the friends of the woman *whose life had been so skillfully saved.* The gentleman of whom this was said claimed to be a respectable physician.

Editors are like all other people in the world (*except physicians*) in this, that whatever they do beyond the requirements of an exact justice to their fellow-men, and an ennobling *amor patriæ*, they do for a consideration; in other words, they are much too shrewd to blow anybody's horn, unless they are furnished the wherewith *to raise the wind.*—*Leavenworth Medical Herald.*

